# Accuracy of 3D volumetric image registration based on CT, MR and PET/CT phantom experiments

**DOI:** 10.1120/jacmp.v9i4.2781

**Published:** 2008-07-09

**Authors:** Guang Li, Huchen Xie, Holly Ning, Deborah Citrin, Jacek Capala, Roberto Maass‐Moreno, Peter Guion, Barbara Arora, Norman Coleman, Kevin Camphausen, Robert W. Miller

**Affiliations:** ^1^ Radiation Oncology Branch National Cancer Institute Bethesda MD U.S.A; ^2^ Department of Nuclear Medicine, Clinical Center National Institutes of Health Bethesda MD U.S.A

**Keywords:** 3D volumetric image Registration, accuracy, multi‐modality imaging, image visualization, radiation treatment planning, image‐guided radiation therapy (IGRT)

## Abstract

Registration is critical for image‐based treatment planning and image‐guided treatment delivery. Although automatic registration is available, manual, visual‐based image fusion using three orthogonal planar views (3P) is always employed clinically to verify and adjust an automatic registration result. However, the 3P fusion can be time consuming, observer dependent, as well as prone to errors, owing to the incomplete 3‐dimensional (3D) volumetric image representations. It is also limited to single‐pixel precision (the screen resolution). The 3D volumetric image registration (3DVIR) technique was developed to overcome these shortcomings. This technique introduces a 4th dimension in the registration criteria beyond the image volume, offering both visual and quantitative correlation of corresponding anatomic landmarks within the two registration images, facilitating a volumetric image alignment, and minimizing potential registration errors. The 3DVIR combines image classification in real‐time to select and visualize a reliable anatomic landmark, rather than using all voxels for alignment. To determine the detection limit of the visual and quantitative 3DVIR criteria, slightly misaligned images were simulated and presented to eight clinical personnel for interpretation. Both of the criteria produce a detection limit of 0.1 mm and 0.1°. To determine the accuracy of the 3DVIR method, three imaging modalities (CT, MR and PET/CT) were used to acquire multiple phantom images with known spatial shifts. Lateral shifts were applied to these phantoms with displacement intervals of 5.0±0.1mm. The accuracy of the 3DVIR technique was determined by comparing the image shifts determined through registration to the physical shifts made experimentally. The registration accuracy, together with precision, was found to be: 0.02±0.09mm for CT/CT images, 0.03±0.07mm for MR/MR images, and 0.03±0.35mm for PET/CT images. This accuracy is consistent with the detection limit, suggesting an absence of detectable systematic error. This 3DVIR technique provides a superior alternative to the 3P fusion method for clinical applications.

PACS numbers: 87.57.nj, 87.57.nm, 87.57.‐N, 87.57.‐s

## I. INTRODUCTION

Radiation therapy has been improved in recent years owing to technical advances, including image‐based treatment planning as well as image‐guided treatment delivery.^(^ ^1^ ^–^ ^5^ ^)^ Multi‐modality imaging techniques that have been routinely applied in radiation treatment planning (RTP) include: computed tomography (CT), magnetic resonance imaging (MR), and positron emission tomography (PET). Through image registration,^(^ ^6^ ^–^ ^10^ ^)^ patient anatomy and physiology can be combined and visualized, providing a comprehensive view of the therapeutic target, together with surrounding normal tissues. The addition of coaxial imaging equipment to megavoltage X‐ray accelerators, including on‐site cone beam CT^(11)^ ^,^ ^(12)^ and Tomotherapy Imaging,^(13)^ ^,^ ^(14)^ has set a new foundation for image‐guided radiation therapy (IGRT) development, by providing immediate pre‐treatment verification and adjustment of a patient's position, resulting in improved accuracy of conformal radiation treatment delivery. This high‐precision radiation treatment delivery (RTD) minimizes normal tissue toxicity and opens a path to more aggressive fractionation schemes. It also permits a transition to frameless intra‐/extra‐cranial stereotactic radiation therapy, with improved patient comfort and clinical outcome.^(^ ^15^ ^–^ ^18^ ^)^ Image registration plays the key role in providing optimum alignment between the pre‐treatment setup image and the planning image,^(^ ^19^ ^–^ ^22^ ^)^ minimizing deviation (patient setup uncertainty) of the RTD from the RTP.

Principal image registration techniques include intensity‐based automatic registration, as well as visual‐based manual registration.^(^ ^6^ ^–^ ^10^ ^,^ ^23^ ^–^ ^29^ ^)^ Automated registration techniques have been used increasingly in RTP and IGRT,^(^ ^13^ ^,^ ^14^ ^,^ ^25^ ^,^ ^27^ ^,^ ^29^ ^)^ based on maximization of mutual information (MMI) of multi‐modal images^(^ ^30^ ^–^ ^32^ ^)^ or grayscale similarity (MGS) for single modality images.^(33)^ ^,^ ^(34)^ However, an automatic registration may carry and propagate systematic errors,^(34)^ reach a sub‐optimal solution,^(16)^ or even fail to achieve a reasonable alignment.^(33)^ Realistically, these phenomena exist because most clinical images contain a certain degree deformation, including motion induced deformation and artifacts, especially in the case of PET imaging. Many deformable registration algorithms have been reported,^(7)^ ^,^ ^(30)^ but they all suffer from a lengthy optimization process and are not yet applicable clinically. Simplified techniques have been reported and applied clinically, such as region‐of‐interest registration,^(6)^ intensity‐weighted registration,^(14)^ and discrete rigid body approximation,^(32)^ but manual adjustment is clinically required based on visual verification, combined with anatomical and physiological knowledge.^(^ ^24^ ^–^ ^28^ ^)^

Prior to the recent development of the 3D volumetric image registration (3DVIR) method,^(27)^ the only viable manual fusion method was based on three orthogonal planar views (3P).^(^ ^6^ ^,^ ^13^ ^,^ ^24^ ^–^ ^28^ ^)^ In addition to “fine‐tuning” automatic registration results, this manual method was also used in establishing initial conditions for an automatic registration and for an independent registration. Because this 3P fusion is based on 2D visualization, it only provides partial 3D information at any given time. A 3D alignment is actually achieved through massive, non‐visual correlation among a series of planar views in three orthogonal directions, resulting in the following major shortcomings: (1) large inter‐/intra‐observer variations,^(26)^ (2) time consuming, tedious methodology,^(^ ^25^ ^–^ ^29^ ^)^ (3) single‐pixel precision,^(^ ^24^ ^,^ ^26^ ^,^ ^27^ ^)^ (4) fewer reliable anatomical landmarks for functional images,^(25)^ and (5) global registration errors.^(27)^ Using the manual 3P fusion technique to verify and adjust the results of an automatic image registration will adversely affect the final registration accuracy, making it both observer‐dependent and error prone.

The 3DVIR technique overcomes most of the shortcomings of the 3P fusion method.^(27)^ This registration technique aligns the image using anatomic structure volumes and surfaces by employing a new dimension, namely the homogeneity of color distribution on an anatomical landmark. Additionally, the criteria associated with the 3DVIR provide instant feedback on the quality of the alignment and offer guidance for further iterations. The method presents a visual volumetric correlation of landmarks, eliminating the repetitive, tedious and observer‐dependent evaluation process inherent in the 3P fusion method. Previously, the 3DVIR technique was compared with MMI‐based automatic registration for cross‐verification.^(27)^

In this study, an improved 3DVIR technique and its validation against experimental data in three imaging modalities will be reported. The technical improvements have included (1) incorporation of image classification to visualize internal registration landmarks in real‐time, (2) introduction of a quantitative registration criterion to further reduce observer dependency, and (3) use of decimal precision in transformation and interpolation for sub‐voxel registration capability. Validation and accuracy assessment of the 3DVIR have been performed based on three phantom imaging experiments (CT, MR and PET/CT), by comparing calculated registration shifts with measured spatial shifts in the phantom position. The detection limit of the 3DVIR has been assessed visually and quantitatively using eight clinical personnel and a plot of quantitative criterion versus spatial shifts, respectively. Both accuracy and detection limit were found to be 1/10 voxel (1 voxel ~ 1 mm) for all three imaging modalities. The advantage of using the 3DVIR technique over the manual 3P fusion method and automatic MMI/MGS registration methods will be discussed in terms of accuracy and reliability, based on the ability to selectively use the most reliable, volumetric registration landmarks.

## II. METHODS

**Figure 1:**
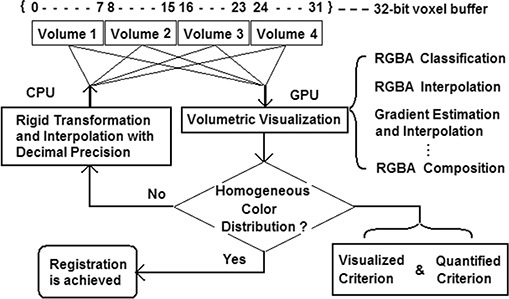
The flow chart of 3D volumetric image registration process. The 4 volumetric image data are stored in a 32‐bit voxel buffer array, which can be retrieved and manipulated in the two different processes: registration transformation and volumetric visualization. The registration process iterates until the registration criterion is satisfied visually and/or quantitatively.

**Figure 2:**
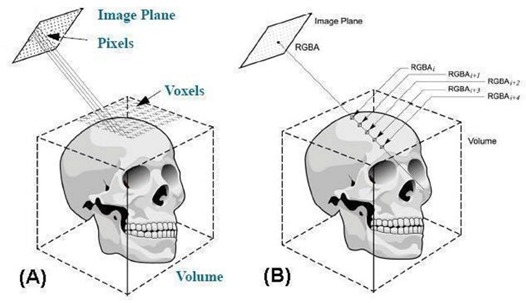
Demonstration of ray‐casting algorithm for volumetric visualization. (a) The relationship of voxels in an image volume and pixels in an image plane and (b) RGBA accumulation along a ray until the accumulated opacity reach unity.

The key of the 3DVIR technique, which was described previously,^(27)^ is the registration criterion, namely the homogeneity of color distributed on a given anatomic landmark, with the registering images represented by pseudo‐mono‐colors, such as red (R), green (G) or blue (B). The volumetric registration flow chart is shown in Fig. 1. The volumetric image visualization is supported by a volume rendering video card for real‐time performance based on the ray‐casting visualization algorithm, as shown in Fig. 2. All registration operations are performed in real‐time, including landmark classification and visualization, registration transformation and interpolation, calculation of the volumetric registration criteria, and viewpoint manipulation.

### A. Volumetric image visualization and registration

#### A.1 RGBA lookup tables (LUTs)

A lookup table (LUT) is a map (transfer function) that associates a set of scalar values (grayscale) to color (R, G, and B) and visibility (opacity: A or Alpha) of a set of data points (voxels). A LUT is often overlaid with image histogram, facilitating the color mapping (a one‐dimensional visualization technique). The opacity LUT (A) overrides the color LUTs (RGB); that is, when a voxel is transparent (A=0) the assigned color does not matter as it becomes invisible. Under the RGBA visualization format, the LUT is a sophisticated version of the “Window/Level” (W/L) control, defining which voxels are visible and what color they are. In other words, the W/L is the simplest case of a LUT with a linear Level function (increasing from zero to unity) within a grayscale Window range.

**Figure 3:**
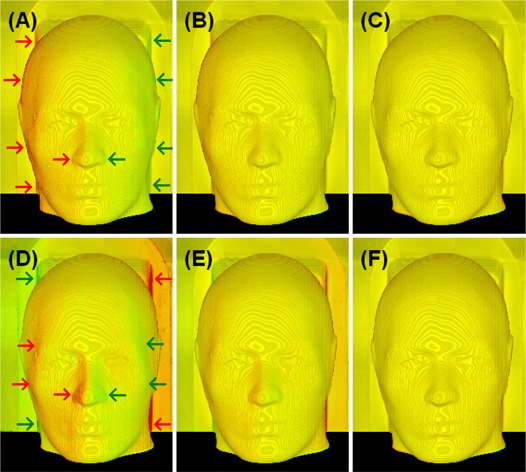
Volumetric views of two identical CT phantom images with simulated spatial shifts. Top row (A to C): translational shifts (Xt) of 0.5, 0.2 and 0.0 voxels (voxel=0.78mm) were applied to the aligned image (C); Bottom row (D to F): rotational shifts (Xr) of 0.5°, 0.2° and 0.0° were applied to the aligned image (F). The color homogeneity on the “skin” landmark improves as the alignment is improved. The translational (lateral) shifts appear mostly on the left and right sides of the image volume: the larger the surface grayscale gradient and the larger the surface oblique angle (between the ray and surface normal), the larger the visual color inhomogeneity would be. The rotational (around the superior‐inferior axis through the center of the image volume) shift causes non‐uniform displacements in the directions perpendicular to the rotational axis: the larger the distance of the viewing voxel to the rotational axis, the bigger the rotational displacement and so the more dramatic color inhomogeneity.

A mono‐colored image can be realized using either a single LUT (such as R, with G=B=0) or identically weighted LUTs (such as white, with R=G=B). A linear color LUT(s) can be used to show the grayscale image in mono‐color for stereoscopic visualization of anatomical “landscape”, as shown in Fig. 3(C). It is worthwhile to note that a mild texture (“iso‐elevation‐contour” pattern) appears due to the unevenness of the volumetric surface caused by limited imaging resolution, as shown in Fig. 3. For the two superimposed images, slightly different colored LUTs can make the existing texture colorful (similar to a diffraction pattern) that should not be misinterpreted as an image misalignment.

Four default LUTs, which are provided by the software for each of the image volumes, can be modified by the user, based on the image histogram, as well as the visualized volume. The criteria for establishing a suitable LUT are based on if a desired anatomical volume is visualized by adjusting Alpha‐LUT. Linear RGB‐LUTs can be used within the A‐Window range that affects the mono‐colored rendition of the grayscale image for optimal stereoscopic visualization. This anatomy‐based visualization does not require a precise LUT function, as long as the registration images have a similar volume. Slight LUT differences may result in a color weighted appearance (producing base line color difference and local colored texture), but will not affect the global homogeneity of the color distribution.

#### A.2 Ray‐casting algorithm

Ray‐casting is an algorithm for image‐ordered volume rendering.^(36)^ The basic idea is to determine the pixel values in an image plane by sending an array of rays through these pixels into the scene based on the current camera settings, such as viewing angle, as shown in Fig. 2(A). For RGBA visualization format, the rays accumulate RGBA values along the way and blend them into the pixel for display, until the accumulated opacity (A) becomes unity (at which point all voxels are opaque), as shown in Fig. 2(B). The mathematical equations for the RGBA accumulation used in this study are discussed in a later section. In fact, a variety of blending functions can be applied to the ray‐casting visualization, generating very different views of the same images, such as the maximum intensity projection (MIP). One major drawback for the ray‐casting visualization is that it is fairly slow and it is necessary to use hardware‐based volume rendering to achieve real‐time performance.^(^ ^37^ ^,^ ^38^ ^)^

#### A.3 Volumetric image registration operations

Volumetric image registration relies on global views of the homogeneity of color distribution within the visualized volume. It emphasizes the use of multiple viewing angles because rigid transformations affect the volumetric alignment in a systematic fashion. Any systematic change in the color distribution of the image volumes reflects their relative alignment and indicates the adjustment required to improve the homogeneity of color distribution. Distinguishing a translational misalignment from a rotational one is straightforward. For instance, a lateral translational shift causes a lateral displacement in the color inhomogeneity. In contrast, an axial rotation (passing through the center of the volume) causes color inhomogeneity that increases radically from the rotational axis (the further the voxel is from the rotational axis, the more dramatic the inhomogeneity, as shown in Fig. 3). In addition, the opposing color biases due to the rotation show not only laterally, but also in any directions perpendicular to the axis of rotation. So, viewing the volume from multiple directions should facilitate distinguishing between a rotational misalignment from a translational one. The facial “landscape” plays a significant role as well in distinguishing between the two different shifts. In the extreme case, a spherical phantom would not provide any information on rotational shifts about its central axis, but a translational displacement will clearly show.

The ability to have multiple views in real‐time is the key to identifying and eliminating any systematic misalignment. For multi‐modality images, it is expected that there will be some local color bias due to contrast, content and resolution differences among imaging modalities. The objective is to look for an overall color distribution inhomogeneity displaying a systematic pattern. We recommend rotational adjustments, followed by translation to superimpose the images. This process iterates until a satisfactory result is obtained.

#### A.4 Four concurrent image registration

The image voxel buffers are designed to permit the registration of up to four concurrent image volumes. Any of the four image volumes can be selectively turned on or off, if there is a need to focus on fewer images. Only one image can be moved (translated or rotated) at any given time. It is recommended to register any two images sequentially, followed by the cross‐verification and cross‐adjustment among all four images. Fundamentally, the three primary colors (RGB) provide the limit in the number of images that can be simultaneously registered with visual tracking. Practically, a tertiary color (white) can be used to represent the fourth image. An ambiguity may be introduced since white voxels can result from either perfect alignment of RGB voxels or the white image, but this can be resolved by turning on and off the white image. In our clinical research, registration of four concurrent images has been performed whenever more than two imaging modalities are involved, including this study. Previously, it was reported that registration of a set of CT, MR (T1), MR (T2) and PET images was performed in a single process for pre‐treatment planning and post‐treatment evaluation.^(35)^ Not only can this single process perform registration of up to four images, but also combine registration with visual verification. The registration of four concurrent images eliminates potential error propagation if only two images are allowed for registration and multiple images have to be registered sequentially.^(27)^

### B. Image classification of anatomic landmarks

#### B.1 Image classification using opacity lookup table (A‐LUT)

Image classification in the 3DVIR technique is achieved through a built‐in opacity (A, or Alpha) value, which is assigned to each voxel through a lookup table, together with three pseudo‐color (R, G, or B) LUTs, as the RGBA visualization format.^(36)^ The A‐LUT operation over the image histogram determines the visibility of the voxel content displayed, while R, G, and B‐LUTs determine the color of the voxel. For any image point with intensity (I), the visible voxel intensity (WI) can be obtained using the RGBA LUTs *if* s) through a vector transformation:

(1) $$VVI=f(I)=\left[\begin{array}{l} f_{R}(I)\\ f_{G}(I)\\ f_{B}(I)\\ f_{A}(I) \end{array}\right]$$

In a CT image, for instance, there are two distinct interfaces with large voxel intensity differences: skin/air and bone/soft‐tissue boundaries. Based on the definition of CT number, these differences are as large as half the grayscale range. Therefore, both interfaces are readily extracted using the opacity LUT, controlled in real‐time by the graphical user interface (GUI). In MR and PET, the skin/air (and brain/bone) interfaces also possess significant intensity differences, in both phantom and patient images. As a matter of fact, skin is one of the few complete anatomies shown in patient PET images.

#### B.2 Visual amplification of the alignment of classified landmarks

The WI is a new dimension beyond the 3D volumetric space, in which image alignment is examined. Because of the large intensity differences at the interface of a selected landmark over a voxel displacement in space, it amplifies the signal in 3D space. As discussed above, skin/air and bone/soft‐tissue interfaces possess very large intensity gradient. Mathematically, it can be expressed as:

(2) $$\begin{array}{l} dVVI=f(dD(x,y,z)),\text{or}\\ \frac{dVVI}{dD}>>1 \end{array}$$

where *dWI* is intensity differential resulting from *dD*, which is a spatial displacement within a voxel (1 voxel ~ 1 mm), and *f()* is an amplification function.

With the introduction of the decimal precision in the transformation and interpolation of the registration, the spatial displacement of images can be a fraction of a voxel. When the image alignment is evaluated volumetrically, any systematic bias in intensity (color) at the landmark will indicate a misalignment, which serves as guidance for further alignment.

### C. Quantitative volumetric registration criterion

#### C.1 Retrieving the visible voxel intensity on an anatomic landmark via ray‐casting

To quantify the visual 3D volumetric registration criterion, the visible voxel must be retrieved in real‐time via a ray‐casting algorithm.^(37)^ ^,^ ^(38)^ A ray in a given viewing direction was cast through the center of a pixel on an image plane, representing one or a series of voxels along the ray in a volume, as shown in Fig. 2. The RGBA values of the voxel points along each ray were accumulated, producing a visible pixel intensity. The penetration depth of the ray (or the thickness of the visible voxel layer on the landmark) was set to shallow, by using a narrow opacity window. The matrix of pixels from an array of parallel rays formed a visual image of the volume.

Quantitatively, the following recursive functions were used for rendering the visible image using front‐to‐back blending (accumulation) of RGBA (colors and opacity):

(3) $$\begin{array}{l} R_{Accum}^{i+1}=R_{Accum}^{i}+(1.0-A_{Accum}^{i})\cdot R^{i}\cdot A^{i}\\ G_{Accum}^{i+1}=G_{Accum}^{i}+(1.0-A_{Accum}^{i})\cdot G^{i}\cdot A^{i}\\ B_{Accum}^{i+1}=B_{Accum}^{i}+(1.0-A_{Accum}^{i})\cdot B^{i}\cdot A^{i} \end{array}$$

(4) $$A_{Accum}^{i+1}=A_{Accum}^{i}+(1.0-A_{Accum}^{i})\cdot A^{i}$$

where *i* and i+1 represent current and next ray depth, respectively. Note: both accumulated opacity $\left(A_{\text{Accum}}^{i}\right)$ and voxel opacity ($A^{i}$) affect the volumetric visualization. When $A_{\text{Accum}}^{i}$. <1.0, a voxel is invisible if its opacity $\left(A^{i}\right)$ equals zero. When $A_{\text{Accum}}^{i}=1.0$, all voxels (>i) are invisible, since they do not contribute to the pixel RGB values (Equation 3).

When registering multiple image volumes, one ray may reach a visible voxel that has contributions from more than one image volume, if they coincide at that particular voxel. Each voxel buffer contains four fields, as shown in Fig. 1, and up to four image volumes can be registered simultaneously. The RGBA‐LUTs affect only the image visualization (which is useful in identifying the first layer of visible voxels) but not the voxel data stored in the voxel buffer. The uniformity of the WI contributions at the surface of the landmark is used in the quantified registration criterion, as discussed below.

#### C.2 Quantitative analysis of the homogeneity of color distribution in real‐time

By definition, the visual homogeneity of the color distribution on a given anatomical landmark should have minimal variance in the visible voxel intensity difference (VVID) between any two mono‐colored imaging modalities. Therefore, for registered images a random color distribution (snow pattern) should be seen on the landmark; whereas a misalignment should appear to have a systematic color‐biased distribution (global alignment aberration), indicative of a systematic spatial displacement.

Uniform sampling across the image plane is used for calculating the criterion, and about 4% of the pixels are sufficient to correctly identify a registration point, while retaining real‐time performance. For any visible voxel (i), the VVID is: $\Delta I_{i}=I_{i}^{A}-I_{i}^{B}$, where $I_{i}^{A}$ and $I_{i}^{B}$ (<256=8 bits) are the WI from images A and B, respectively. For all sampled voxels, the variance of the VVID can be expressed as:

(5) $$VAR=\sum_{i=1}^{N}\frac{{\left(\Delta I_{i}-\Delta I\right)}^{2}}{N}=\sum_{i=1}^{N}\frac{{\left(I_{i}^{A}-I_{i}^{B}-\Delta I\right)}^{2}}{N}$$

where $\Delta I=\sum \left(\Delta I_{i}/N\right)$ 0<q<1 q<0 q>1 0<q<1 represents the average of the VVID and N is the total number of pixels sampled, excluding completely transparent rays. In the case of two identical images, the variance of VVID decreases as the image alignment improves, approaching zero with a perfect alignment.

In multi‐modality image registration, the average voxel intensity of an anatomical landmark can differ dramatically. Owing to high baseline differences between two given modalities, the VAR value can become insensitive to the VVID. The sensitivity is substantially improved by incorporating a weighting factor (R):

(6) $$R=\frac{I^{A}}{I^{B}}=\frac{\sum_{i=1}^{N}I_{i}^{A}/N}{\sum_{i=1}^{N}I_{i}^{B}/N}=\frac{\sum_{i=1}^{N}I_{i}^{A}}{\sum_{i=1}^{N}I_{i}^{B}}$$

and the Equation (5) is modified, producing an intensity‐weighted variance:

(7) $$mVAR=\sum_{i=1}^{N}\frac{{\left(\Delta I_{i}^{*}-\Delta I^{*}\right)}^{2}}{N}=\sum_{i=1}^{N}\frac{{\left((I_{i}^{A}/R)-I_{i}^{B}-\Delta I^{*}\right)}^{2}}{N}$$

where $\Delta I*=\sum \left(\Delta I_{i}*/N\right)$ is the average of modified VVID $\left(\Delta I_{i}*=I_{i}^{A}/R-I_{i}^{B}\right)$. This quantitative measure, when minimized, indicates an optimal registration, which can be independently verified by visual examination, avoiding local minima.

### D. Tests for quantitative and visual detection limit of the registration criteria

Two identical CT images (red and green) with simulated rotational or translational shifts were used to evaluate the quantitative and visual detection limit of the 3DVIR criteria, using the variance analysis and eight independent observers. Superimposition of the images produced a yellow image, due to the color blending of equally weighted red and green contributions, as shown in Figs. 3(C) and 3(F). When one of the two images was slightly rotated or translated, the misalignment produced an inhomogeneous color distribution, as shown in Figs. 3(A), 3(B), 3(D) and 3(E).

For the quantitative test, incremental image shifts of 0.1° and 0.1 voxel, relative to the registration point, were simulated and used. The VAR value was calculated in different views for each of the image shifts. These values were plotted resulting in a well‐shaped curve.

For the visual test, eight observers were asked to identify color biases in 12 images, which contained various shifts of 0.0, 0.1 and 0.2 units (degrees or voxels; 1 voxel=0.78mm) in any of the six degrees of freedom. These images were randomly presented to the observers as a slide show. A correct determination scored one point and an incorrect one scored zero. Statistical analysis of the results yielded a visual detection limit for the 3DVIR criterion.

### E. Head phantom positioning and imaging with pre‐determined translational shifts

#### E.1 Head phantom positioning accuracy

In all three imaging studies, the phantoms were immobilized with a head holder. Graph paper with 0.13 mm lines (1 mm grid), which were verified using a reference line, were taped on the scanner couches and on four sides of the phantom holder. A magnification glass was used for the line alignment at each side. The phantom was displaced at a regular interval of 5.0±0.1mm between scans. The positioning uncertainty was limited by the width of the gridline (<0.13mm). The room lasers were not employed for phantom alignment and displacement, because the width of their projected line exceeded the desired alignment accuracy. Typically, four positions with three lateral shifts were used for image acquisition.

Lateral uncertainty of the couch position was negligible (within ±0.1mm) since it is not movable laterally. Longitudinally, however, the couch positioning uncertainty of <±0.5 mm (based on the manufacturer's specification) was bigger than the gridline width (0.13 mm), dominating the longitudinal uncertainty. Therefore, the lateral comparisons were used for the accuracy evaluation of translational alignment. Experiments with rotational shifts were not conducted due to difficulties in accurate phantom setup using the graph papers, as well as the unavailability of an accurate shifting device for rotation. However, the rotational registration accuracy evaluation using experimental data remains our interest and will be examined in the future.

All images were pre‐processed automatically by tri‐linear interpolation to have an image size of 320×320 and an isotropic voxel size (~ 1 mm, varying with modalities, see below) with an 8 bit grayscale, prior to registration. For multi‐modality image registration, the image field of view was kept the same for both modalities.

#### E.2 CT head phantom image acquisition

**Figure 4:**
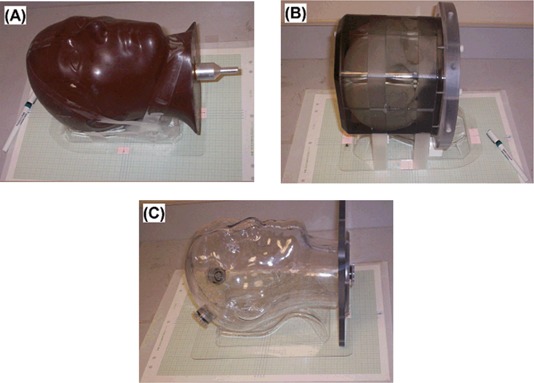
Three head phantoms for (A) CT, (B) MR and (C) PET/CT experiments. Head holders and tapes were used to immobilize the phantoms, and graph papers and magnifying glass were used for phantom positioning. The finest line width of 0.13 mm was used to align two gridlines on the couch and the phantom holder. The alignment was checked on all four sides of the phantom holder for a translational shift.

A tissue equivalent anthropomorphic head phantom with internal structures, as shown in Fig. 4(A), was scanned using a CT scanner (AcQSim, Philips Medical Systems). Sixteen images were acquired in an array of positional offsets (lateral and longitudinal), having 5.0±0.1mm displacement intervals. The original voxel size was 0.49×0.49×2.0 mm (with an original image size of 512×512), and the reformatted voxel size was 0.78×0.78×0.78 mm (in the final image size of 320×320).

#### E.3 MR head phantom image acquisition

A water‐based head phantom with internal structures, as shown in Fig. 4(B), was scanned in a 1.5T MRI scanner (Intera, Philips Medical Systems). Four axially‐scanned images were acquired with lateral shift intervals of 5.0±0.1mm. The images were processed to exclude the geometrically‐shaped external voxels outside the skull. The “brain” image, as defined by the inner surface of the skull, was extracted for registration. The original voxel size was 0.90×0.90×90.2mm (with an original image size of 256×256), and the reformatted voxel size was 0.72×0.72×72.0mm (in the final image size of 320×320).

#### E.4 PET/CT head phantom image acquisition

An anthropomorphic head phantom filled with F18‐fluoro‐deoxy‐glucose ^(18^F‐FDG) solution (1 mCi), shown in Fig. 4(C), was scanned using a combined PET/CT scanner (DiscoveryST, GE Healthcare). The thickness of the phantom wall was approximately 3−4mm. Four image sets were acquired with lateral shift intervals of 5.0±0.1mm. The original PET voxel size was 4.25×4.25×25.3mm (with an original image size of 128×128) and the original CT voxel was 0.98×0.98×98.3mm (with an original image size of 512×512). Some empty space in the field of view was trimmed and the reformatted voxel size for both PET and CT images was 1.0×1.0×0.1mm (in the final image size of 320×320).

#### E.5 Alignment of the PET/CT scanner

The PET/CT scanner alignment was determined using a solid rod phantom (${\;}^{68}\text{Ga}/{\;}^{68}\text{Ge}$), 9.5 mm in diameter and 312 mm in length. The rod was placed with its axis parallel to the direction of couch motion and normal to the image plane. The rod image was arbitrarily divided into three segments, which were used as independent measures. In both PET and CT images, the identical region of interest was applied and the centers‐of‐mass (or centers‐of‐activity) were calculated for comparison. Because the rod was uniform in activity and density, the center of mass should be coincident with the center of geometry. Therefore, the difference between the two centers was indicative of the alignment quality of the combined‐modality scanner.

#### E.6 Expression of the Registration Accuracy

The accuracy of image registration is stated with its precision using standard deviation. The unit of accuracy can be expressed in degree for rotational shifts and in voxels and/or in millimeters (mm) for translational shifts. In physical space, by definition, a voxel is the smallest unit of an image volume, so its size should carry a volumetric unit, such as mm^3^. In image space, however, a voxel is a dot (often isotropic) with an assigned grayscale, and is used as a unit length for any image operation. For the sake of simplicity, the lengths of a cubic (voxel) edge in mm are frequently used to describe the voxel size. A linear scaling relationship exists between voxel and mm, such as 1.0 voxel=0.78 mm in CT, 1.0 voxel=0.72 mm in MR, and 1.0 voxel=1.0 mm in PET/CT. Here, we have used either voxel and mm, or mm alone, as the unit for image transformation and registration accuracy, as well as in comparison with previously reported results. Clinically, the units of mm and degree are more preferable.

## III. RESULTS

### A. Quantitative measure of the 3DVIR criterion

**Figure 5:**
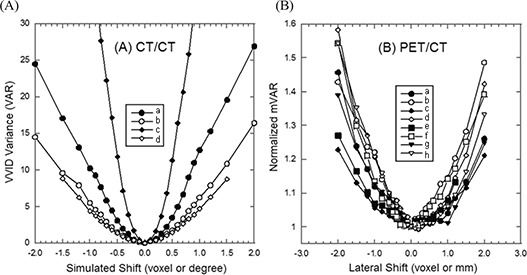
Quantitative criteria vs. spatial shifts (translation or rotation). (A) Identical CT/CT image alignment: VAR criterion is used in lateral direction (Xt, translation) or lateral axis (Xr, rotation). Legends: (a) anterior and (b) superior views of Xt translational shifts, and (c) anterior and (d) superior views of Xr rotational shifts. (B) Co‐registered PET/CT image alignment: mVAR criterion is used in lateral direction (Xt, translation). From (a) to (h), eight curves of the 4 set PET/CT images based on superior views (solid symbols) and anterior views (open symbols) are shown.

Fig. 5(A) shows the quantitative measure (VAR) of color homogeneity versus rotational or translational shifts in the lateral direction with increments of 0.1° or 0.1 voxel (0.08 mm). Two identical images superimposed perfectly at the registration point, resulting in a null variance of VVID (VAR in Equation 5) and uniform color homogeneity (Fig. 3(C). The variance increases exponentially with relative image displacement, forming a well shaped curve. This is consistent with the color inhomogeneity increase, as shown in Fig. 3. The curve slope is steeper in the anterior view than the superior view, suggesting that the detection sensitivity is higher due to greater anatomic “landscape” details.

**Figure 6:**
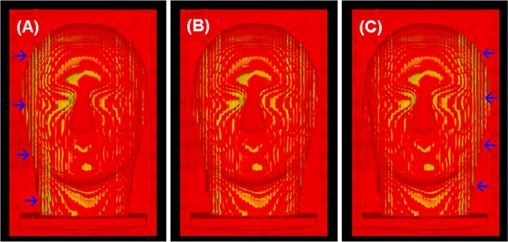
Anterior views of PET/CT images with lateral off‐alignments: (A) −0.5 voxels (mm), (B) 0.0 voxel (mm) and (C) +0.5mm voxels (mm). The arrows point to the region with color inhomogeneity. Note: the local color inhomogeneity shown in (B) is caused by different imaging resolutions in PET and CT and slightly different R/G‐LUTs on the two image histograms.

Fig. 5(B) shows normalized curves from four, co‐registered PET/CT images using the modified VVID variance (mVAR in Equation 7). These curves demonstrate an excellent agreement (0.05±0.09mm or voxel) between the hardware and software registration, indicating that the mVAR provides an accurate measure of the quality of PET/CT registration. The lateral alignment of the combined PET/CT scanner was determined to be <±0.1 mm. Again, the slope of the well curves is steeper in the anterior than in the superior view. Comparing with CT/CT registration curves in Fig. 5(A), the curves in Fig. 5(B) are much shallower, indicating a relatively low sensitivity of the “skin” voxel alignment in PET/CT images. Fig. 6 shows the anterior views of three PET/CT images with shifts of −0.5mm, 0.0 mm and +0.5mm, relative to the co‐registration point. The accuracy is independent of imaging modalities because the registration criteria are built in the 4th dimension beyond the 3D image space.

### B. Visual detection limit of the 3DVIR criterion

Table 1 shows the results for determination of the visual detection limit. Under the experimental conditions, two coincident color images were correctly identified by observers as homogeneous (Fig. 3(C) with a success rate of 94%. For images misaligned by 0.2 voxels (0.16 mm) of translation or 0.2° of rotation (as illustrated by Figs. 3(B) and 3(E), the color inhomogeneity was identified with a 100% success rate by all eight observers. When the misalignment was reduced to 0.1° and 0.1 voxels (0.08 mm), inconsistency started to occur. However, the average success rate was still 80%. Interestingly, the two lateral shifts (δXr and δXt in Table 1) had a 100% success rate, suggesting that the results were dependent upon image orientation and could be improved by providing additional volumetric views. Therefore, the visual detection limit for identifying color inhomogeneity using skin as the volumetric landmark was determined to be 0.1° and 0.1 voxel.

**Table 1:** Detection limit of the 3D volumetric image registration criterion (visual). Two identical CT images were used to generate 12 images with various simulated spatial shifts in anterior view, including 2 perfectly aligned images. Eight clinical observers were participated the test: a correct identification of color homogeneity or inhomogeneity scores one point, while a failure scores zero. Here 1.0 voxel=0.78 mm for the CT images.

*Observer*	*Spatial Shifts (0.2° or 0.2 voxel)*						*Spatial Shifts (0.1° or 0.1 voxel)*						*Aligned*
	*δXr*	*δYr*	*δZr*	*δXt*	*δZt*	*Σ/5*	*δXr*	*δYr*	*δZr*	*δXt*	*δZt*	*Σ/5*	*Σ/2*
1	1	1	1	1	1	**1.0**	1	1	0	1	1	**0.8**	**1.0**
2	1	1	1	1	1	**1.0**	1	1	1	1	1	**1.0**	**1.0**
3	1	1	1	1	1	**1.0**	1	1	1	1	1	**1.0**	**1.0**
4	1	1	1	1	1	**1.0**	1	1	0	1	1	**0.8**	**1.0**
5	1	1	1	1	1	**1.0**	1	0	1	1	1	**0.8**	**0.5**
6	1	1	1	1	1	**1.0**	1	0	1	1	1	**0.8**	**1.0**
7	1	1	1	1	1	**1.0**	1	0	1	1	0	**0.6**	**1.0**
8	1	1	1	1	1	**1.0**	1	0	1	1	0	**0.6**	**1.0**
Average						**1.0**						**0.80**	**0.94**
St. Dev.						**0.00**						**0.18**	**0.15**

### C. Accuracy of CT/CT phantom image registration

Table 2 shows the result of the CT/CT phantom image registration accuracy as: 0.03±0.12 voxels (0.02±0.09mm) in lateral (x) direction and 0.33±0.27 voxels (0.27±0.21mm) in longitudinal (z) direction. This variation was attributed to the longitudinal uncertainty (<±0.5 mm) in the couch positioning (movable), while the lateral uncertainty of the couch (unmovable) was negligible. Therefore, the lateral comparison provides the best measure of registration accuracy, yielding a value of ~ 0.1 mm, which is similar to the experimental accuracy of phantom positioning. As a by‐product, this phantom experiment using 3DVIR can provide a quality assurance assessment of couch mechanical accuracy.

**Table 2:** Comparison of the CT phantom translational shifts and CT/CT registration shifts. The phantom “skin” was used as the 3D volumetric image registration landmark, and the visual color homogeneity was used as registration criterion. The registration shifts were calculated by physical distance (mm)=(voxel shift)×(voxel size), where 1.0voxel=0.78mm. The registration was cross‐confirmed using the “skull” as landmark. The uncertainty of the CT couch positioning was specified within ±0.5mm in longitudinal direction and within ±0.1mm in lateral direction by the manufacturer. The phantom positioning had ±0.1mm uncertainty by aligning reference lines of 0.13 mm in width under optical amplification.

*Experimental Shifts (mm)*		*Registration Shifts (mm)*				*Statistical Analysis (mm)*		
Lateral	$X_{\text{Exp}}$	$X_{1}$	$X_{2}$	$X_{3}$	$X_{4}$	$X_{\text{Avg}}$	$X_{\text{Exp}}$‐$X_{\text{Avg}}$	St. Dev.
	5.0±0.1	4.92	4.92	4.99	5.07	4.98	**0.02**	**0.08**
	10.0±0.1	9.92	10.14	9.99	9.99	10.01	−0.01	**0.09**
	15.0±0.1 Average	14.91	14.91	14.91	15.08	14.95	**0.05**	**0.10**
	Average						**0.02**	**0.09**
Longitudinal	$Z_{\text{Exp}}$	$Z_{1}$	$Z_{2}$	$Z_{3}$	$Z_{4}$	$Z_{\text{Avg}}$	$Z_{\text{Exp}}-Z_{\text{Avg}}$	St. Dev.
	5.0±0.5	5.00	4.68	4.61	4.68	4.74	**0.26**	**0.04**
	10.0±0.5	9.76	9.53	9.92	9.59	9.70	**0.30**	**0.21**
	15.0±0.5	14.84	14.52	15.15	14.44	14.74	**0.26**	**0.39**
	Average						**0.27**	**0.21**

**Figure 7:**
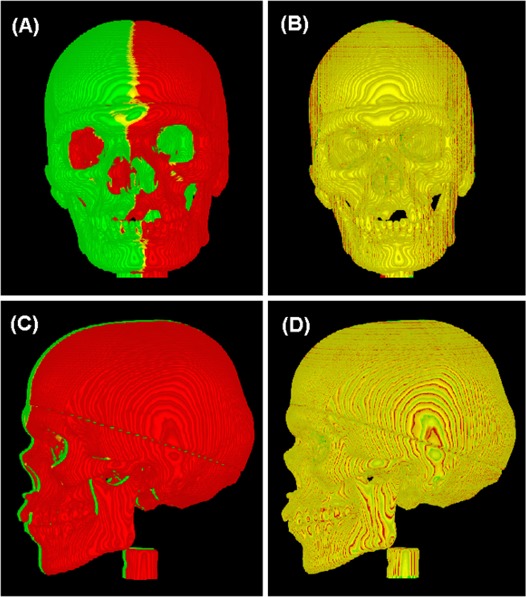
Using bony anatomy as registration landmark. The original two CT images (A, C) are 15.0±0.1mm apart laterally and the registered images (B, D) are obtained with a lateral shift of 15.00 mm. A minor superior‐inferior shift of 0.3 voxel (0.23 mm) is made to compensate the couch positioning error. Note: the local color inhomogeneity (contour pattern) shown in (B, D) is caused by limited imaging resolution and visualization with slightly different R/G‐LUT settings, rather than global image misalignment.

Fig. 7 shows the CT/CT registration using bony landmarks, which were visualized by changing the A‐LUT interactively. Using the independent bony landmark, the 3DVIR registration accuracy remains unchanged, suggesting that the internal and external landmarks are equally reliable in the phantom image registration. This finding provides flexibility in the selection of landmarks as well as the ability to cross‐verify registration of rigid images. Bony anatomy can be used as a more reliable landmark when motion or deformation of soft tissue is present.

### D. Accuracy of MR/MR phantom image registration

Table 3 shows the results of the MR/MR image registration accuracy using 4 images shifted laterally by 5.0±0.1mm intervals. The accuracy was found to be 0.04±0.10 voxels (0.03±0.07mm), using the phantom “brain” (inner skull) as the registration landmark. In MR images, the brain interface is intact since there is a natural grayscale change at the boundary with the skull, which possesses void voxel. This can serve as another internal landmark for 3DVIR, with an accuracy consistent with that of the CT/CT registration.

**Table 3:** Comparison of the MR phantom translational shifts and MR/MR registration shifts. The phantom “brain” voxels (confined by the skull) were extracted by image segmentation and used as the 3D volumetric registration landmarks. The visual color homogeneity was used as registration criterion. Here 1.0 voxel=0.72 mm for the MR images.

*Experimental Shift (mm)*	*Registration Shift (mm)*			*Statistical Analysis (mm)*	
Lateral Shift ($X_{\text{Exp}}$)	$X_{1}$	$X_{2}$	$X_{3}$	$X_{\text{Exp}}$‐$X_{\text{Avg}}$	St. Dev.
5.0±0.1	5.03	4.96	5.10	0.03	0.07

### E. Alignment of the PET/CT scanner and co‐registered PET/CT images

The agreement between the center of mass for CT and PET images of the rod phantom was 0.05±0.13mm laterally and 0.00±0.18mm vertically. Therefore, the alignment of the combined PET/CT scanner was determined to be within ~ 0.1 mm in the image plane.

Four co‐registered PET/CT images were used to validate the quantitative mVAR criterion using the “skin” landmark, as shown in Figs. 5(B) and 6. The agreement between the experimental co‐registration and the mVAR curve prediction was found to be within 0.1 voxel (mm). The PET “skin” volume was defined using the CT volume as reference, and the presence of the thin phantom wall did not affect the registration.

### F. Accuracy of PET/CT, PET/PET and CT/CT phantom image registration

Table 4 shows the registration accuracy for PET/CT images to be 0.03±0.35 voxels (mm) using visual criterion, and 0.05±0.09 voxels (mm) using the quantified (mVAR) criterion. Both results show similar accuracy, but higher precision using the quantitative criterion. Due to the low quality of the PET image, the quantitative measure produces a more reliable indication than the visual judgment.

**Table 4:** Comparison of the PET/CT phantom translational shifts and PET/CT registration shifts. The phantom superficial voxels were used as the 3D volumetric registration landmarks and the visual color homogeneity was used as registration criterion. The registration shift of $X_{\text{AB}}$ represents the lateral distance between two adjacent pairs of PET and CT images with 5.0 mm apart, where A and B are the numbers (1−4) of CT and PET images, respectively, in the 4 sets of PET/CT images. Here 1.0 voxel=1.0 mm for the reformatted PET and CT images.

*Experimental Shift (mm)*	*Registration Shift (mm)*						*Statistical Analysis (mm)*	
Lateral Shift ($X_{\text{Exp}}$)	$X_{12}$	$X_{21}$	$X_{23}$	$X_{32}$	$X_{34}$	$X_{43}$	$X_{\text{Exp}}$‐$X_{\text{Avg}}$	St. Dev.
5.0±0.1	4.5	5.5	5.2	4.8	5.0	4.8	0.03	0.35

Using the same image sets, the accuracies of CT/CT and PET/PET registration were found to be 0.04±0.10 voxels (mm) and 0.09±0.31 voxels (mm) respectively, using the visual criterion, similar to previous results.

## IV. DISCUSSION

### A. Accuracy and reliability of single and dual modality image registration

The registration results of CT/CT, MR/MR, PET/PET and PET/CT images show an excellent agreement between physical shifts in phantom position and corresponding image registration shifts, resulting in an overall accuracy of 0.1 voxel (~ 0.1 mm), independent of imaging modality. In most cases, multiple volume surfaces serve as landmarks for the 3D registration, providing a mechanism for rapid cross‐verification. Using both quantitative and visual criteria, together with both superficial and internal landmarks, the volumetric registration offers a more reliable and versatile tool for clinical use.

A separate assessment of rotational accuracy was not performed experimentally due to difficulty in accurate determination of rotational shifts experimentally and dependency of the result upon the location of the rotational axis. Using simulated image sets with rotational shifts, a visual detection limit of 0.1° was shown. Clinically, a 0.1° rotation may cause >0.1 (voxel) displacement at the surface, since the center of volume is used as the center of rotation. For instance, assuming a head with ~200 mm separation, a 0.1° rotation will result in a voxel displacement of R×sinθ=100×sin0.1°=0.17mm at the surface. This is above the detectable limit (0.1 voxel, which is ~ 0.1 mm in this study). For larger anatomies, such as torso, the bigger radical distance from the center of volume should produce larger spatial shift at the surface. So, an accuracy of 0.1° for rotational alignment was estimated.

The 3DVIR criteria are built into the VVID dimension beyond 3D space, resulting in a 0.1 voxel (mm) detection limit and accuracy. This phenomenon is resulted from the amplified projection from 3D space to the VVID space (Equation 2), so that a small spatial shift can result in a large color difference. In the cases of skin and bony landmarks, the interface has high contrast (spanning half of image grayscale), enabling detection of fractional voxel misalignment, as illustrated in Figs. 3 and 7. Note that the subtle texture (“iso‐elevation contours”) on the image results from the limited imaging resolution (particularly 2 mm slice thickness);^(36)^ such mono‐colored or colored local visual effects (or artifacts) can be eliminated using uniform RGB‐LUT settings. This volumetric visualization knowledge is useful in distinguishing local visual artifacts (the volume surface is not sufficiently smooth, but composed of multiple facets) from a systematic bias in color distribution (due to misalignment).

### B. Applicability of the 3DVIR accuracy to clinical patient image registration

As indicated above, the accuracy of the 3DVIR technique relies upon three key factors: (1) rigid image assumption, (2) volumetric alignment criteria and (3) visualized external and/or internal anatomical landmarks. Two major factors prevent direct translation of the accuracy from this phantom study to clinical patient image registration: (1) patient motion and (2) organ deformation. In the presence of small, random, and rigid motion, which is restricted by using an immobilization device during image acquisition, the 3DVIR can tolerate small volume increases (due to blurring) by readjusting the A‐LUT to achieve a volume match between images. For patient PET/CT images, the PET skin, although low quality, is one of the few complete anatomic landmarks identifiable and can be employed as a volumetric landmark for PET/CT image registration. In the presence of organ deformation, the use of soft‐tissue landmarks will likely introduce a systematic uncertainty. However, the 3DVIR technique allows registration using motion‐free bony landmarks, as shown in Fig. 7. Therefore, the registration accuracy remains unchanged by using stable bony landmarks based on this study. In general, due to the uncertainties in patient setup and patient motion, the image registration accuracy may be reduced, but it should roughly remain in sub‐voxel (sub‐mm) scale. Therefore, this volumetric registration is potentially useful in IGRT patient setup with minimal motion interference, as well as for frameless intra‐/extra‐cranial stereotactic radiosurgery/radiotherapy.

### C. Comparison of the 3DVIR technique with the 3P fusion method

The 3P fusion is based on three orthogonal 2D views of two image volumes at any given time. In order to derive 3D information, all slices in the three orthogonal directions must be viewed sequentially, and reviewed every time the image alignment is adjusted. Additionally, the synthesis of 3D information is dependent upon the cognitive ability of any given observer. Therefore, it is both time consuming and error prone.^(^ ^24^ ^–^ ^27^ ^)^ It is also limited to single pixel precision.

In contrast, the 3DVIR technique reconstructs and visualizes entire image volumes for the observer, who can evaluate the alignment “on‐the‐fly”. The quantitative criterion can be employed to further minimize user dependency, especially in the fine tuning stages. More profoundly, the 0.1 mm accuracy holds not only for the registration of anatomical images, but for functional images as well. The 3DVIR technique presents volumetric images in such a way that the alignment process is reduced to simply merging two objects in “virtual reality” (without perspective visualization). As reported previously, the 3DVIR takes only about one‐third the time required by the 3P fusion to achieve a registration.^(27)^ By using an automatic registration for a pre‐alignment, the performance of the 3DVIR can be further enhanced by at least a factor of three.

### D. Comparison of the 3DVIR technique and MI‐based automatic registration method

Most automatic (rigid) image registration methods based purely on voxel intensity uses all voxels in the fused images, including those of moving organs. Therefore, this form of registration has self‐imposed limitations, due to motion artifacts and deformation of soft tissues caused by respiratory, cardiac, digestive and muscular motion. Additionally, an automatic registration based on this methodology, although reproducible, may contain systematic errors.^(34)^ Therefore, a visual‐based manual fusion is always required to verify and often required to adjust the automatic registration results utilizing specific clinical knowledge.^(13)^ It was reported that the 3DVIR possesses similar registration accuracy as the MI‐based registration for cranial images,^(27)^ so the 3DVIR can be used to evaluate and adjust the automatic result without reducing the overall registration accuracy.

Recently, efforts have been made to incorporate segmentation information into automatic image registrations, by filtering the images so as to alter their voxel weights.^(14)^ The success rate of prostate registration was improved from 65% to 83% by eliminating high contrast voxels (air and bone) using grayscale filters.^(33)^ Semi‐automatic registration with the assistance of manually generated anatomical contours was reported to be useful.^(19)^ The “hybrid” image registration combined with segmentation and visualization has become a trend in pursuing better image registration, especially deformable image registration.^(6)^ ^,^ ^(39)^

The 3DVIR technique registers anatomical landmarks, which are extracted by image classification prior to visualization. These selectively classified landmarks can be more reliable than other voxels, especially when bony landmarks are employed, since they are rigid, well‐defined, and motion‐free (such as the spine). When soft tissues are rendered transparent, the organ motion and deformation can be ignored. Based on the quantified registration criterion, implementation of an automatic registration is the next logical step.

### E. Future direction: a potential semi‐automatic 3DVIR technique

The current 3DVIR technique provides both visual and quantitative registration criteria, which uniquely combine to minimize user dependency. In contrast, none of the visual based manual image registration methods has such a feature, but depends solely upon a user's visual judgment.^(^ ^25^ ^–^ ^29^ ^)^ The real‐time variance analysis provides a tool that is helpful in determining subtle differences in color homogeneity in the “fine tuning” of the registration, while the visual criterion provides both verification and visual guidance throughout the registration process. For multiple modalities, various VVI levels may affect the color baseline, but not the color distribution. This provides a statistical basis for the use of mVAR as a highly sensitive registration indicator (Equation 7). Multiple volumetric views from a variety of angles are helpful to view the global registration volumes. This quantitative criterion provides a foundation for future semi‐automatic registration algorithms. The registration landmark selection and classification must be done manually, while “fine tuning” of a coarse manual 3DVIR alignment can be performed automatically.

## V. CONCLUSION

The accuracy of 3D volumetric image registration of CT, MR and PET/CT images was found to be 0.1 voxel (~ 0.1 mm) and estimated to be 0.1°, based on the three phantom studies. Both superficial (skin) and internal (bone and brain) voxels were found to be suitable as volumetric landmarks. The quantitative registration criterion was found to be as effective as the visual criterion for registration, but provided a higher degree of precision. The capability of using both visual and quantitative measures makes the 3DVIR technique an effective, reliable, and accurate tool for the clinical use. The intrinsic classification and visualization provided by this technique allows registration of bony landmarks, while eliminating interference from organ motion and deformation. In the future, this quantitative criterion can be employed to produce a semi‐automatic 3D volumetric image registration.

## ACKNOWLEDGEMENT

The authors are grateful to Frank Baker and Jan Hardenbergh (TeraRecon Inc.) for their technical support on the volume rendering broad, and to William Dieckmann (Clinical Center, NIH) for performing the data analysis of the PET/CT scanner alignment.

## References

[1] Elshaikh M , Ljungman M , Ten Haken R , Lichter AS . Advances in radiation oncology. Annu Rev Med. 2006;57:19–31. 16409134 10.1146/annurev.med.57.121304.131431

[2] Mackie TR , Kapatoes J , Ruchala K , et al. Image guidance for precise conformal radiotherapy. Int J Radiat Oncol Biol Phys. 2003;56(1):89–105. 12694827 10.1016/s0360-3016(03)00090-7

[3] Xing L , Thorndyke B , Schreibmann E , et al. Overview of image‐guided radiation therapy. Med Dosim. 2006;31(2):91–112. 16690451 10.1016/j.meddos.2005.12.004

[4] Jaffray D , Kupelian P , Djemil T , Macklis RM . Review of image‐guided radiation therapy. Expert Rev Anticancer Ther. 2007;7(1):89–103. 17187523 10.1586/14737140.7.1.89

[5] Li G , Citrin D , Camphausen K , et al. Advances in 4D medical imaging and 4D radiation therapy. Technol Cancer Res Treat. 2008;7(1):67–81. 18198927 10.1177/153303460800700109

[6] Maintz JBA , and Viergever MA . A survey of medical image registration. Med Image Anal. 1998;2(1):1–36. 10638851 10.1016/s1361-8415(01)80026-8

[7] Pluim JP , Maintz JBA , and Viergever MA . Mutual information‐based registration of medical images: a survey. IEEE Trans Med Imag. 2003;22(8):986–1004. 10.1109/TMI.2003.815867 12906253

[8] Hutton BF and Braun M . Software for image registration: algorithms, accuracy, efficacy. Semin Nucl Med. 2003;33(3):180–192. 12931320 10.1053/snuc.2003.127309

[9] Crum WR , Griffin LD , Hill DL , Hawkes DJ . Zen and the art of medical image registration: correspondence, homology, and quality. Neuroimage. 2003;20(3):1425–1437. 14642457 10.1016/j.neuroimage.2003.07.014

[10] Slomka PJ . Software approach to merging molecular with anatomic information. J Nucl Med. 2004;45(1 Suppl):36S–45S. 14736834

[11] Sidhu K , Ford EC , Spirou S , et al. Optimization of conformal thoracic radiotherapy using cone‐beam CT imaging for treatment verification. Int J Radiat Oncol Biol Phys. 2003;55(3):757–767. 12573763 10.1016/s0360-3016(02)04152-4

[12] Pouliot J , Bani‐Hashemi A , Chen J , et al. Low‐dose megavoltage cone‐beam CT for radiation therapy. Int J Radiat Oncol Biol Phys. 2005;61(2):552–560. 15736320 10.1016/j.ijrobp.2004.10.011

[13] Forrest LJ , Mackie TR , Ruchala K , et al. The utility of megavoltage computed tomography images from a helical tomotherapy system for setup verification purposes. Int J Radiat Oncol Biol Phys. 2004;60(5):1639–1644. 15590196 10.1016/j.ijrobp.2004.08.016

[14] Welsh JS , Lock M , Harari PM , et al. Clinical implementation of adaptive helical tomotherapy: a unique approach to image‐guided intensity modulated radiotherapy. Technol Cancer Res Treat. 2006;5(5):465–479. 16981789 10.1177/153303460600500503

[15] Timmerman RD , Kavanagh BD , Cho LC , Papiez L , Xing L . Stereotactic body radiation therapy in multiple organ sites. J Clin Oncol. 2007;25(8):947–952. 17350943 10.1200/JCO.2006.09.7469

[16] Nagata Y , Matsuo Y , Takayama K , et al. Current status of stereotactic body radiotherapy for lung cancer. Int J Clin Oncol. 2007;12(1):3–7. 17380434 10.1007/s10147-006-0646-6

[17] Kavanagh BD , Timmerman RD . Stereotactic radiosurgery and stereotactic body radiatoin therapy: An overview of technical considerations and clinical applications. Hematol Oncol Clin North Am. 2006;20(1):87–95. 16580558 10.1016/j.hoc.2006.01.009

[18] Fuss M , Salter BJ , Rassiah P , Cheek D , Cavanaugh SX , Herman TS . Repositioning accuracy of a commercially available double‐vacuum whole body immobilization system for stereotactic body radiation therapy. Technol Cancer Res Treat. 2004;3(1):59–67. 14750894 10.1177/153303460400300107

[19] Court LE , Dong L . Automatic registration of the prostate for computed‐tomography‐guided radiotherapy. Med Phys. 2003;30(10):2750–2757. 14596313 10.1118/1.1608497

[20] Smitsmans MHP , Wolthaus JWH , Artignan X , et al. Automatic localization of the prostate for on‐line or off‐line image‐guided radiotherapy. Int J Radiat Oncol Biol Phys. 2004;60(2):623–635. 15380600 10.1016/j.ijrobp.2004.05.027

[21] Clippe S , Sarrut S , Malet C , Miguet S , Ginestet C , Carrie C . Patient setup error measurement using 3D intensity‐based image registration technique. Int J Radiat Oncol Biol Phys. 2003;56(1):259–265. 12694847 10.1016/s0360-3016(03)00083-x

[22] Letourneau D , Martinez AA , Lockman D , et al. Assessment of residual error for online cone‐beam CT‐guided treatment of prostate cancer patients. Int J Radiat Oncol Biol Phys. 2005;62(4):1239–1246. 15913917 10.1016/j.ijrobp.2005.03.035

[23] West J , Fitzpartick JM , Wang MY , et al. Comparison and evaluation of retrospective intermodality brain image registration techniques. J Comput Assist Tomogr. 1997;21(4):554–568. 9216759 10.1097/00004728-199707000-00007

[24] Fitzpatrick JM , Hill DLG , Shyr Y , West J , Studholme C , Maurer CR J . Visual assessment of the accuracy of retrospective registration of MR and CT images of the brain. IEEE Trans Med Imaging. 1998;17(4):571–585. 9845313 10.1109/42.730402

[25] Pfluger T , Vollmar C , Wismuller A , et al. Quantitative comparison of automatic and interactive methods for MRI‐SPECT image registration of the brain based on 3‐dimensional calculation of error. J Nucl Med. 2000;41(11):1823–1829. 11079489

[26] Vaarkamp J . Reproducibility of interactive registration of 3D CT and MR pediatric treatment planning head images. J Appl Clin Med Phys. 2001;2(3):131–137. 11602009 10.1120/jacmp.v2i3.2606 PMC5726038

[27] Li G , Xie H , Ning H , et al. A novel 3D volumetric voxel registration technique for volume‐view‐guided image registration of multiple imaging modalities. Int J Radiat Oncol Biol Phys. 2005;63(1):261–273. 16024179 10.1016/j.ijrobp.2005.05.008

[28] Sarkar A , Santiago RJ , Smith R , Kassaee A . Comparison of manual vs. automated multimodality (CT‐MRI) image registration for brain tumors. Med Dosim. 2005;30(1):20–24. 15749007 10.1016/j.meddos.2004.10.004

[29] Ceylan C , van der Heide UA , Bol GH , Lagendijk JJ , Kotte AN . Assessment of rigid multi‐modality image registration consistency using the multiple sub‐volume registration (MSR) method. Phys Med Biol. 2005;50(1):N101–N108. 15876660 10.1088/0031-9155/50/10/N01

[30] Mattes D , Haynor D , Vesselle H , Lewellen , Eubank W . PET‐CT image registration in the chest using free‐form deformation. IEEE Trans Med Imag. 2003;22(1):120–128. 10.1109/TMI.2003.809072 12703765

[31] Cizek J , Herholz K , Vollmar S , Schrader R , Klein J , Heiss WD . Fast and robust registration of PET and MR images. Neuroimage, 2004;22(1):434–442. 15110036 10.1016/j.neuroimage.2004.01.016

[32] Walimbe V , Shekhar R . Automatic elastic image registration by interpolation of 3D rotations and translations from discrete rigid‐body transformations. Med Image Anal. 2006;10(6):899–914. 17079184 10.1016/j.media.2006.09.002

[33] Smitsmans MHP , de Bois J , Sonke JJ , et al. Automatic prostate localization on cone‐beam CT scans for high precision image‐guided radiotherapy. Int J Radiat Oncol Biol Phys. 2005;63(4):975–984. 16253772 10.1016/j.ijrobp.2005.07.973

[34] Guckenberger M , Meyer J , Wilbert J , et al. Cone‐beam CT based image‐guidance for extracranial stereotactic radiotherapy of intrapulmonary tumors. Acta Oncol. 2006;45(7):897–906. 16982556 10.1080/02841860600904839

[35] Li G , Xie H , Ning H , et al. A clinical application of volume‐view‐guided image registration of four concurrent imaging modalities: CT/MR1/MR2/PET. Int J Radiat Oncol Biol Phys. 2005;63(2):S138–S139. 10.1016/j.ijrobp.2005.05.008 16024179

[36] Schroeder W , Martin K , Lorensen B . The visualization toolkit – an object‐oriented approach to 3D graphics. 3rd ed. Kitware Inc; 2004.

[37] Pfister H , Hardenbergh J , Knittel J , et al. The VolumePro real‐time ray‐casting system. Proceedings of the 26th annual conference on Computer graphics and interactive techniques (ACM SIGGRAPH). ACM Press;1999:251–260.

[38] Wu Y , Bhatia V , Lauer H , Seiler L . Shear‐image order ray casting volume rendering. Proceedings of the 2003 symposium on interactive 3D graphics. Monterey, CA: ACM Press; 2003:152–162.

[39] Li G , Citrin D , Miller RW , et al. 3D and 4D medical image registration combined with image segmentation and visualization. In Encyclopaedia of Healthcare Information Systems. Wickramasinghe N. and Geisler E. (Eds), IGI Global, Hershey, PA, 2008.

